# β-Lactoglobulin's Conformational Requirements for Ligand Binding at the Calyx and the Dimer Interphase: a Flexible Docking Study

**DOI:** 10.1371/journal.pone.0079530

**Published:** 2013-11-08

**Authors:** Lenin Domínguez-Ramírez, Elizabeth Del Moral-Ramírez, Paulina Cortes-Hernández, Mariano García-Garibay, Judith Jiménez-Guzmán

**Affiliations:** 1 Departamento de Ciencias de la Salud, Universidad Autónoma Metropolitana-Lerma, Lerma de Villada, Lerma, México; 2 Departamento de Ciencias de la Alimentación, Universidad Autónoma Metropolitana-Lerma, Lerma de Villada, México; 3 Departamento de Biotecnología, Universidad Autónoma Metropolitana, Iztapalapa, Mexico City, Mexico; University of South Florida College of Medicine, United States of America

## Abstract

β-lactoglobulin (BLG) is an abundant milk protein relevant for industry and biotechnology, due significantly to its ability to bind a wide range of polar and apolar ligands. While hydrophobic ligand sites are known, sites for hydrophilic ligands such as the prevalent milk sugar, lactose, remain undetermined. Through the use of molecular docking we first, analyzed the known fatty acid binding sites in order to dissect their atomistic determinants and second, predicted the interaction sites for lactose with monomeric and dimeric BLG. We validated our approach against BLG structures co-crystallized with ligands and report a computational setup with a reduced number of flexible residues that is able to reproduce experimental results with high precision. Blind dockings with and without flexible side chains on BLG showed that: i) 13 experimentally-determined ligands fit the calyx requiring minimal movement of up to 7 residues out of the 23 that constitute this binding site. ii) Lactose does not bind the calyx despite conformational flexibility, but binds the dimer interface and an alternate Site C. iii) Results point to a probable lactolation site in the BLG dimer interface, at K141, consistent with previous biochemical findings. In contrast, no accessible lysines are found near Site C. iv) lactose forms hydrogen bonds with residues from both monomers stabilizing the dimer through a claw-like structure. Overall, these results improve our understanding of BLG's binding sites, importantly narrowing down the calyx residues that control ligand binding. Moreover, our results emphasize the importance of the dimer interface as an insufficiently explored, biologically relevant binding site of particular importance for hydrophilic ligands. Furthermore our analyses suggest that BLG is a robust scaffold for multiple ligand-binding, suitable for protein design, and advance our molecular understanding of its ligand sites to a point that allows manipulation to control binding.

## Introduction

**Figure 1:**
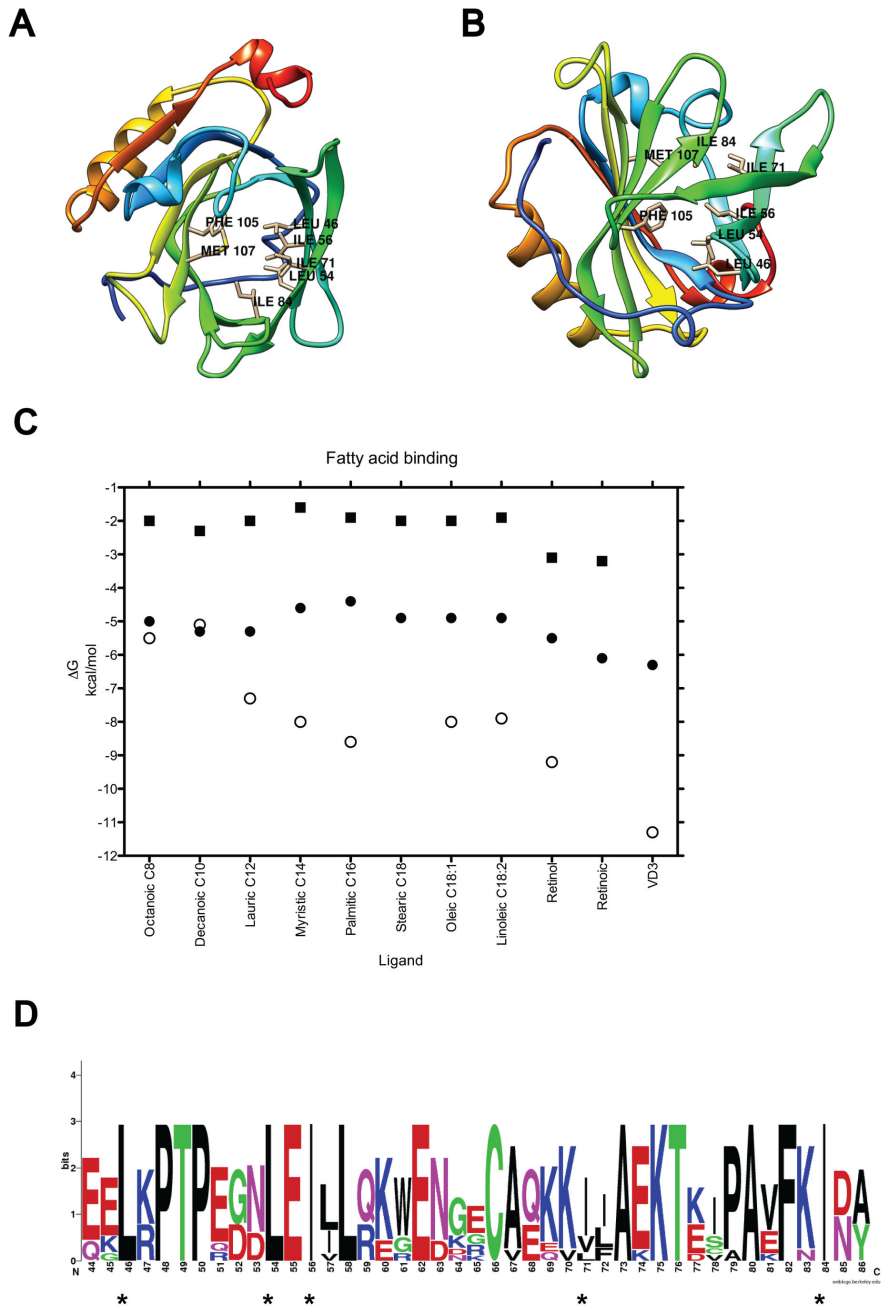
β-lactoglobulin and its calyx binding site. The main BLG binding site (calyx or Site A) is shown empty from two perspectives: (**A**) top-down view and (**B**) bottom-up view. The seven flexible residues required for ligand binding are labeled. The secondary structure is colored from N-terminus in blue, to C-terminus in red. (**C**) Plot of binding energy calculated from docking vs ligand using rigid, monomeric, empty BLG structures with open (2BLG, black circles), or semi closed (2Q39, black squares) EF loops, and compared to experimentally determined data (open circles). Fatty acids are sorted by increasing size, or in the case of stearic, oleic and linoleic, by decreasing saturation. No experimental affinity has been reported for stearic or retinoic acids. (**D**) Weblogo of the sequence alignment of BLG from 7 mammals. Asterisks indicate the 5 residues made flexible for docking.

Bovine β-lactoglobulin (BLG) is an abundant milk protein, making up to 50% of whey and 12% of whole cow milk proteins [1]. It belongs to the lipocalin family composed of small extracellular proteins capable of binding hydrophobic ligands [2-7]. Each BLG monomer consists of 162 residues (18.3 kDa) folded into eight stranded antiparallel β-sheets that form a hydrophobic pocket or calyx, flanked on one side by an α-helix [8] (Figure 1A and B). Although its biological function is uncertain [9], BLG is relevant to the food and pharmaceutical industries due to its ability to bind fatty acids, vitamins and peptides and it has been the subject of numerous biochemical and structural studies. For hydrophobic ligands two sites have been postulated: one inside the calyx (referred here to as Site A) and the other at the dimer interface, on the outer surface of the protein between the α-helix and the β-barrel (hereby referred to as Site B) [5,10]. While both are supported by X-ray crystallography, the calyx is favored. The accessibility to the calyx is pH-dependent and mediated by the mobile EF loop, (residues I84 to N90, Figure S1): when E89 is protonated the loop remains closed and it opens upon deprotonation [11]. NMR data indicate that hydrogen bonds between residues I84, N90, E108 in loops EF and GH modulate EF loop opening [12]. All the structures with ligands bound to the calyx exhibit an open EF loop, suggesting that at neutral pH this site is accessible.

BLG's oligomeric state also changes as a function of pH, a phenomenon known as Tandford transitions [11,13,14]. At room temperature and pH below 4.0 and above 5.2, the protein consists predominantly of monomers and dimers. Despite wide acceptance that BLG exists mainly as a dimer in cow’s milk (pH 6.8 and high protein concentration) [13,15], binding to the dimer hasn’t been thoroughly explored. BLG dimers have received little attention even when discussing X-ray determined (XRD) structures that display a dimerization interface with a ligand bound [5]. We and other authors [15-17] posit that the dimer is the relevant BLG form for ligand binding in biological scenarios. To explore this hypothesis and to better understand the binding requirements of each BLG site, we implemented a computational approach. Blind molecular docking of the BLG monomer and dimer with 13 known ligands was performed and validated against the XRD structures. We analyzed the binding requirements of each known site in terms of side chain flexibility, residue conservation and polarity; then explored the binding of lactose.

Lactose is the most abundant BLG ligand in milk, making up to 4.8% of cow's milk, versus only 0.1% of other sugars [18-20]. However, lactose's binding site in BLG remains undefined as they haven’t been co-crystalized and there is little evidence to unambiguously discern if it can bind the same sites as hydrophobic molecules. Lactose chemically modifies lysine residues in BLG, in a slow reaction (t1/2 for 1 lactose per BLG is about 35 hours at 60° C, [21]) termed lactolation or lactosylation. Lactolation sites suggest accessible docking sites for the sugar [22]. We evaluated BLG's putative binding sites for lactose in our validated computational set up and report here that *in silico*, lactose binds the dimer interface and an alternative site C present in the monomer, but does not bind the calyx.

## Materials and Methods

### Three-Dimensional Modeling

Three-dimensional bovine β-lactoglobulin models, monomer and dimer, were obtained from the molprobity website [23]. We used the XRD coordinates of bovine BLG, isoform A, PDB ID 2BLG from a crystal grown without ligands at pH 8.2 and with loop EF in its open conformation [11]. This empty structure was used for docking to avoid ligand bias. Four other structures were used as docking controls or for RMSD (root mean square deviation) analyses: 1BSY [11], another empty structure with open EF loop; 1B0O [2], with palmitic acid bound to the calyx and open EF loop; 2Q39 [24], empty and with a semi-open EF loop and 3BLG [11], empty and with the EF loop in a closed conformation. These proteins were crystallized at pH 7.1, 7.5, 7.4 and 6.8 respectively. Hydrogen atoms and charges were added using Autodock Tools [25]. The BLG structures co-crystallized with ligands were obtained from the RCSB [26] and are enumerated in Table 1. Most of them are isoform B. Illustrations were prepared using UCSF Chimera [27], PyMol (DeLanoScientific, 2009) and Weblogo [28]. 

**Table 1:** Experimental and theoretical details of monomeric BLG binding sites.

**Ligand**	**Octanoic acid**	**Decanoic acid**	**Lauric acid**	**Myristic acid**	**Palmitic acid**	**Bromo decanoic**	**Stearic acid**	**Oleic acid**	**Linoleic acid**	**Retinol**	**Retinoic acid**	**Vitamin D3^a^**	**DDM**
**MW**	*144.2*	*172.2*	*200.3*	*228.3*	*256.4*	*279.2*	*284.8*	*282.4*	*280.4*	*286.4*	*300.4*	*384.6*	*510.6*
**Ligand-protein contacts^b^**	8 / 0	23 / 0	16 / 0	21 / 0	21 / 2	15 / 0	19 / 0	28 / 1	19 / 0	19/1	23 / 0	23 / 0	11 / 1
**Exp. binding energy kcal mol^-1^**	-5.5	-5.1	-7.13	-8.0	-8.6	NA	NA	-8.0	-7.9	-9.2	NA	-11.3	NA
**Rigid binding energy^c^ kcal mol^-1^**	-5.0	-5.3	-5.3	**-4.6**	**-4.4**	**-4.6**	**-4.9**	**-4.9**	**-4.9**	**-5.5**	**-6.1**	**-6.3**	**-5.7**
**Binding energy with 5 flexible residues^d^ kcal mol^-1^**	-6.0	-6.4	-6.8	-7.0	-7.2	-6.6	-7.4	-7.6	-7.6	-6.4	-8.5	**-6.1**	-8.4
**Binding energy with 7 flexible residues^e^ kcal mol^-1^**	-6.0	-6.6	-7.1	-7.1	7.3	-6.7	-7.6	-7.9	-8.0	-7.3	-9.3	-9.1	-7.9
**Isoform**	B	B	B	B	B	A	B	B	B	B	B	B	B
**PDB**	3NQ9[6]	3NQ3 [6]	3UEU [41]	3UEV [41]	3UEW [41]	1BSO [45]	3UEX [41]	4DQ3^f^	4DQ4 ^g^	1GX8 [3]	1GX9 [3]	2GJ5 [5]	2R56 [46]

a Data for binding to the monomer.

b As determined by ligplot from XRD structures. Can also be obtained from the PDBSUM website. First number is apolar contacts, second is polar contacts.

c Receptor kept rigid in the original 2BLG conformation.

d Energies calculated from docking to 2BLG with residues L46, L54, I56, I71 and I84 set as flexible.

e Docking to 2BLG with residues L46, L54, I56, I71, I84, F105 and M107 set as flexible.

f Superseeds 3QZK, to be published.g Superseeds 3QZJ, to be published.

NA stands for Not Available

Binding energies in bold correspond to dockings to site C, the only site found in those cases. All others are dockings to Site A.

### Molecular Docking

Blind docking was performed using Vina 1.1.2 [29] on a 12-core computer running Mac OS X. All ligands were obtained from the ZINC database [30] and converted to PDBQT format using the GUI provided by Autodock Tools [25]. This was done to avoid bias derived from starting with structurally known XRD BLG-ligand complexes. Ligands were checked manually against the known chemical structure and all their rotatable bonds (angles chemically allowed to rotate) remained free from restraint during docking. The receptor for docking was kept rigid except where noted. Blind docking employed a 1 Å grid size and grid dimensions of 46 x 46 x 46 centered on the monomer or 70 x 70 x 70 centered on the dimer. Exhaustiveness was always set to 1000. All our dockings have been reproduced at least twice. Three repetitions have been preformed for central experiments in the paper. Analysis of the docking results was performed in PyMOL (DeLanoScientific, 2009) as well as in Seelinger’s Autodock/Vina plugin [31]. When flexible residues were used on BLG all side chain bonds were allowed to rotate except for the Cα -Cβ. 

### Computational analysis

Secondary structure assignments were made using DSSP v2.0.4 [32] and visualization was via PyMol through the DSSP plugin provided by Zhu, H [33]. Ligplot [34] was employed locally to dissect the interactions between BLG and its ligands. Ligplot is also accessible at the PDBSUM website [35]. Naccess [36] and hbplus [37] were used to quantify solvent accessibility and H-bond number, respectively. 

## Results

### Dissection of the known binding site in monomeric bovine β-lactoglobulin

BLG binds most of its XRD ligands into the hydrophobic pocket or calyx (Site A), located at the center of its β-barrel (Figure 1A and B), without any apparent requirement for a dimeric arrangement. The only exception, as shown by XRD structure 2GJ5 [5], is the bulky ligand vitamin D3 (VD3) that binds both the calyx and the dimer interface. The Cα -RMSD between 2BLG, empty and with an open lid to the calyx (open EF loop) and 13 structures with open lids and ligands bound to the calyx is low, ranging from 0.37 to 1.2 Å (Figure S1C). Notably, in this global alignment most conformational changes cluster in loops EF and GH, located at the mouth of the calyx and previously reported as mobile [12,38]. To further explore the local flexibility in relation to calyx accessibility, we performed pairwise RMSD alignments between 2BLG and three structures in different states: open/empty (1BSY), closed/empty (3BLG) and open/palmitic bound (1B0O). Again, most conformational changes are circumscribed to loops at the calyx entrance, in particular EF (Figure S2). These analyses suggest that loop flexibility is crucial for calyx accessibility, in accordance with previous reports [12,39,40].

Next, we quantified the BLG residues in contact with 13 different ligands and the type of contact in XRD structures bound to fatty acids, detergents and vitamins (Table 1 and Table S1), to get a global overview of the ligand binding reported in literature. We found 23 ligand binding residues, located to the calyx's β-sheets (Figure 1A and B). Interestingly, all binding residues and in fact, the entire β-barrel that forms the calyx, sustain minimal movement in global (Figure S1C) and pairwise RMSD analyses (Figure S2), suggesting that calyx backbone flexibility is not required for binding. As an approach to test this idea and elucidate the calyx's conformational determinants for binding we decided to test whether an open EF loop was enough for BLG structures to bind, *in silico*, the 13 XRD ligands in blind docking. We first tested empty BLG structures with open (2BLG) or semiclosed (2Q39) EF loops, presented as rigid, monomeric receptors. No ligand was able to bind the calyx in the semi-closed receptor (2Q39), underlining the importance of calyx accessibility for ligand binding. Binding energies were distinctively low for the semiclosed 2Q39 (Figure 1C, squares), and all ligands located to another site, hereby referred to as Site C, located between sheet A and the last helix before the C-terminus**.**

**Figure 2:**
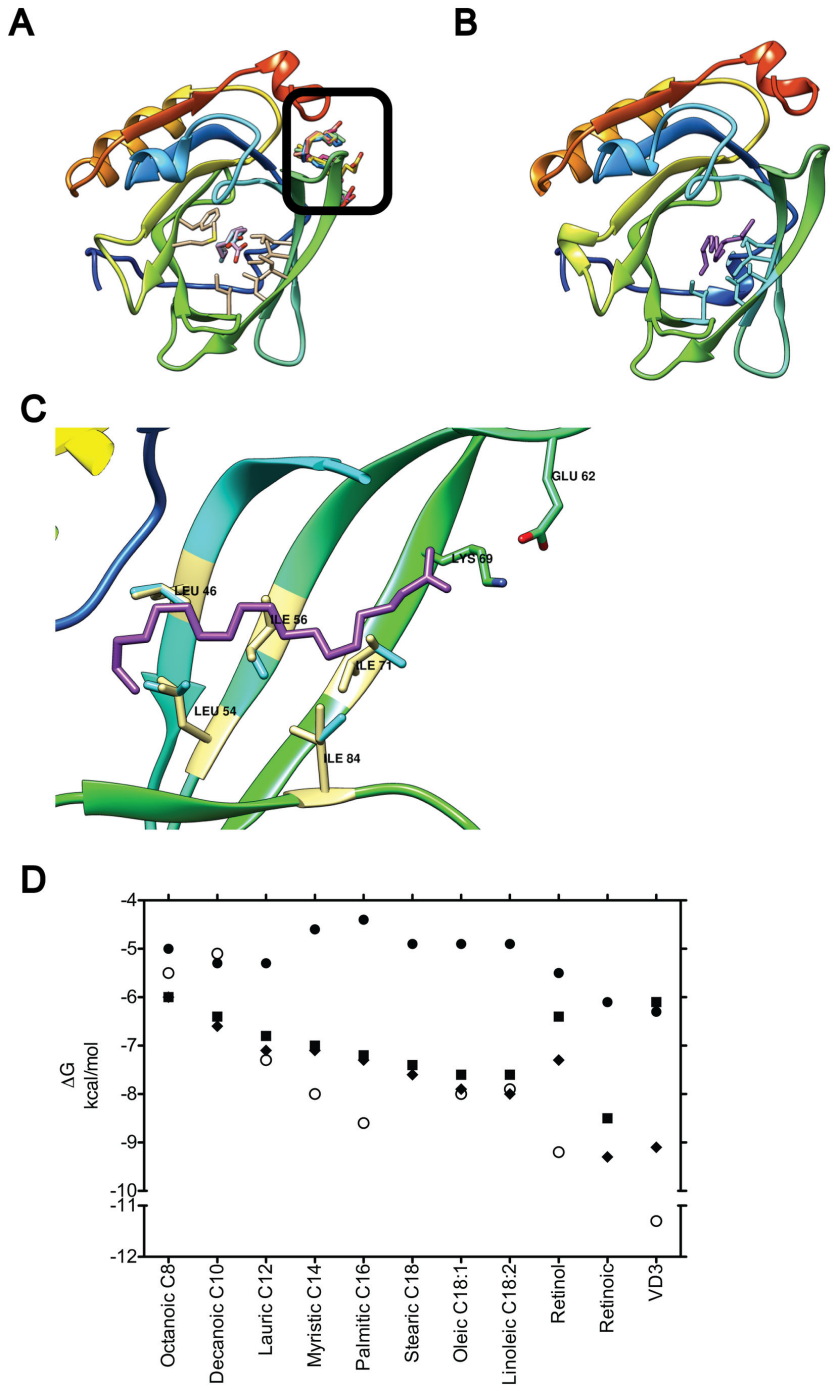
Effect of residue flexibility on fatty acid binding. (**A**) Docking to a rigid 2BLG allows only the three smallest lipids into the calyx (shown superposed in white, light blue and light purple), while excluding longer fatty acids to Site C (black square, fatty acids in different colors). The seven binding residues in the calyx are shown in light yellow. When five of these residues were allowed flexibility all fatty acids bind the calyx. Stearic acid (purple) is shown bound in (**B**) and (**C**) in a full BLG top down view and a side view magnification of the calyx, respectively. In (**C**) the five flexible residues (blue) are shown aligned to their XRD counterpart (light yellow) to highlight movements that enable docking. (**D**) Plot of binding energy from docking vs. ligand using the monomeric empty, 2BLG, either rigid (black circles) or with 5 (black squares) or 7 (black triangles) flexible residues. Experimentally determined energies are shown for comparison (open circles). Fatty acids are sorted as in Figure 1C.

On the open rigid receptor (2BLG) only the three smallest ligands docked inside the calyx with affinities comparable to the experimentally determined, namely: octanoic acid (-5 kcal mol^-1^), decanoic acid (-5.3 kcal mol^-1^), and lauric acid (-5.3 kcal mol^-1^) (listed by increasing molecular weight in Table 1 and Figure 1C). Longer lipids were not able to insert into this site, but located to Site C with low affinities (> -5.3 kcal mol^-1^, Table 1). These results prompted us to test whether allowing movement of specific side chains among the binding residues (see methods) was enough to enable known ligands to fit the calyx. To determine the minimal number of flexible residues required for binding we selected residues in contact with the ligand in at least six XRD structures and with the highest count of ligand interactions. We initially selected five residues: L46, L54, I56, I71 and I84 (Figure 1A and 1B, Table S1), highly conserved across ungulates (Figure 1D). Although residues F105 and M107 filled the requirements too, they were initially set aside due to their bulkiness. By allowing the initial five residues to move their side chains, 12 of the tested ligands can bind Site A in the open receptor (2BLG) with energies between -6 and -8.5 kcal mol^-1^, comparable to the experimentally determined (Table 1 and Figure 2D). A typical result is shown in Figure 2B and C: docking of stearic acid requires the slight rotation of the five side chains selected. As in the XRD structure, stearic acid bends to fit the pocket. Remarkably, residues E62 and K69, experimentally determined to contact this ligand, were found at a similar distance from the stearic acid both through docking and XRD [6,41]. Once flexibility of these five residues is allowed, binding of fatty acids to the calyx of 2BLG shows almost a linear correlation between increasing fatty acid size and binding energy (Figure 2D). In contrast, side chain flexibility in the calyx of the semi-closed receptor (2Q39) does not result in ligand binding (not shown).

**Figure 3:**
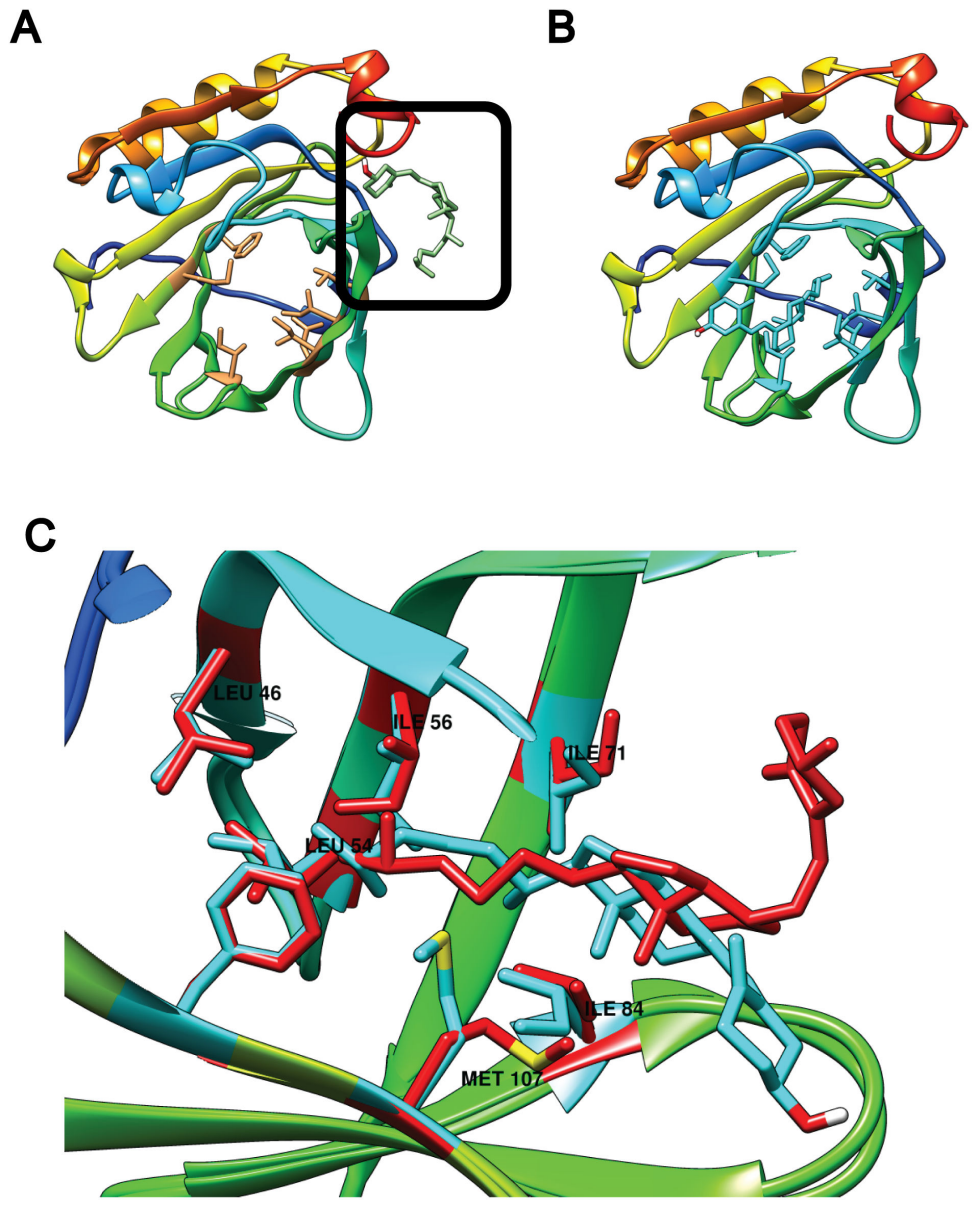
Effect of residue flexibility on VD3 binding to monomeric BLG. (**A**) Docking to 2BLG with a rigid calyx locates VD3 to Site C (black square). In contrast, when seven residues are rendered flexible, VD3 fits in the calyx (**B** and **C**). In (**C**) a side view magnification of the calyx compares the VD3 docking result (blue) and the residues made flexible (blue), to their XRD counterparts (red).

Retinoic acid and retinol were also able to bind the calyx in 2BLG with only five flexible residues but VD3 was not (Figure 3A). However, adding flexibility to the largest residues in our list, F105 and M107, for a total of seven flexible residues was enough to allow vitamin D3 binding at the calyx with high affinity (Table 1, Figure 3B). Interestingly, to allow for VD3 binding the flexible side chains moved only modestly (Figure 3C), just as with fatty acids. When compared with the XRD structure obtained in the presence of VD3, only M107 shows a very different conformation. Another contrast is that only the VD3 region that lies inside the calyx is similar between our docking and the XRD (Figure 3C). The VD3 region protruding seems to be less restricted, as expected, since it is facing the solvent. The use of seven flexible residues also improved retinol and retinoic acid binding by up to 0.9 kcal mol^-1^ (Table 1), but didn't change fatty acid binding (Table 1 and Figure 2D). Our results suggest that the empty but rigid calyx in 2BLG, even when accessible due to the open lid, is not in the correct conformation for ligand binding. Yet a conformation that permits binding is reached by allowing flexibility only in the side chains of a limited set of calyx residues. To further test this idea we used PDB 1B0O [2,42], a BLG structure determined in the presence of palmitic acid, where the binding site is already formed. Binding energies were close to experimental for fatty acids when docking to 1B0O. Moreover, using the same five or seven flexible residues did not further increase the binding energies (Figure S4), likely because the conformation that allows binding was already attained in the crystal.

Thus, our computational setup, namely docking to 2BLG with seven flexible residues, behaves in terms of ligand binding as does the monomeric BLG in biochemical and crystallographic experiments. Furthermore, we have found the minimal calyx flexibility requirements for all known crystallographic ligands to bind in the known site. However, none of these conditions (rigid protein, five or seven flexible residues) allowed lactose to bind inside the calyx. Thus, we next explored ligand binding through blind docking to potential sites on the dimeric BLG. To undertake this, we first validated our setup with VD3, the only ligand known to bind the interface. 

### Vitamin D3 Binding to Bovine β-Lactoglobulin in Dimeric State

**Figure 4:**
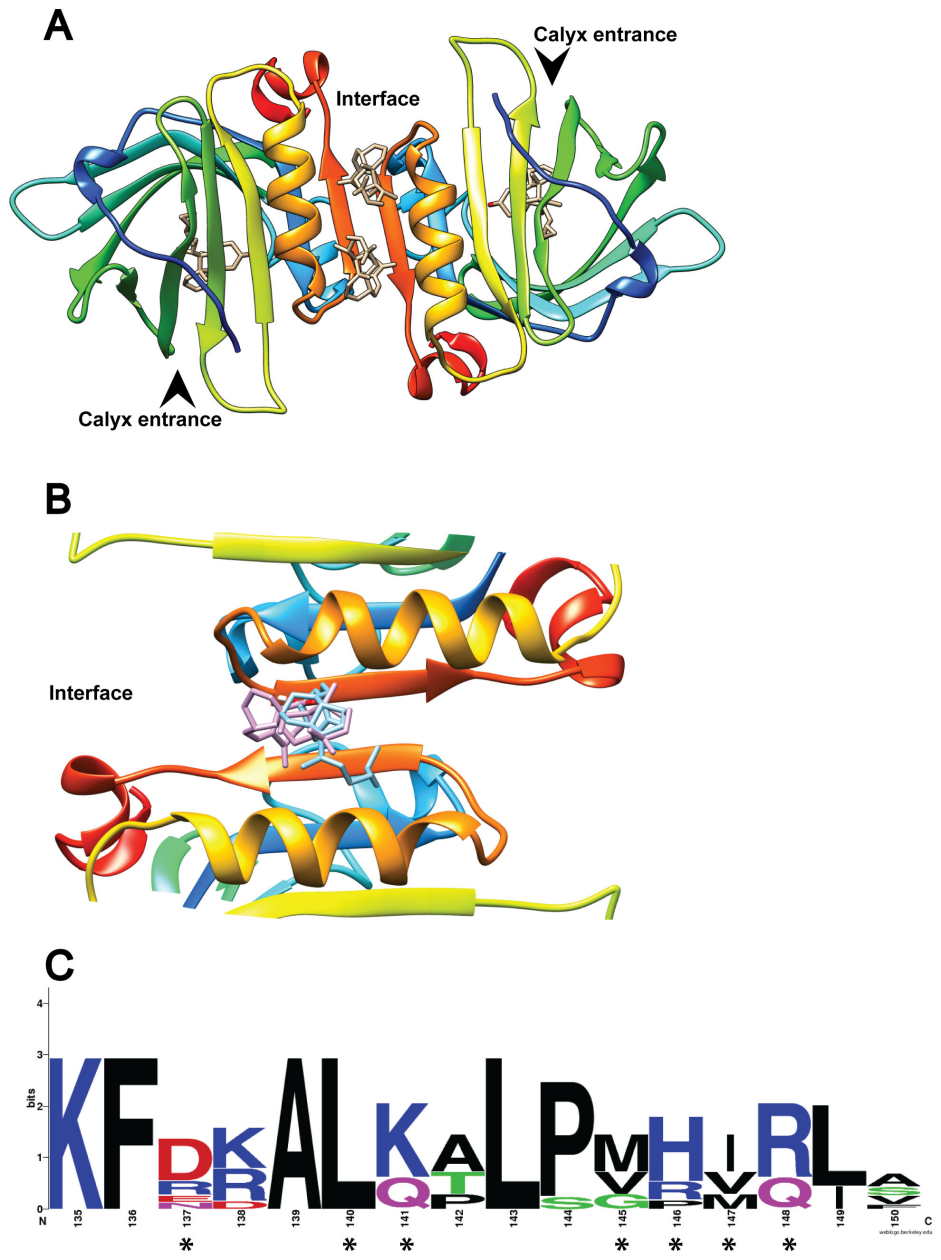
VD3 binding to the BLG dimer interface. (**A**) The BLG dimer is shown with the four experimentally determined VD3 ligands at their respective binding sites. The arrowheads indicate entrance to the calyx. In (**B**) only one experimental VD3 (purple) is compared to the best docking result obtained with flexible residues (“full interface” in 2BLG, in blue). (**C**) Shows the weblogo of 7 ungulate BLG sequences highlighting the residues that bind VD3 with asterisks.

For VD3 two binding sites have been shown by crystallographic (PDB 2GJ5) and biochemical data [5,43]: the calyx (Site A) and the dimer interface (Site B). When a BLG dimer is formed by crystallographic symmetry from PDB 2GJ5, two VD3 molecules are present per dimer interface (Figure 4A). The nature of this interfacial-binding site is dramatically different from the calyx: each monomer contributes with four hydrophilic residues that, while not fully conserved, do show a tendency towards polarity (Figure 4C). The interfacial average accessible area per residue per monomer is 29%, while in the calyx it is 8.5%, making the interface favorable to hydrophilic ligands. 

We first tested whether VD3 could successfully dock at the interfacial site in the rigid 2BLG: blind docking showed a remarkable affinity for the rigid Site B (Table 2 and Figure 4B). To check the effect of residue flexibility on binding at the interface, two subsequent flexible docking strategies were employed using 2BLG. First, we defined a "half interface" set-up where the seven residues that bind VD3 at the interface of each BLG monomer (D137, L140, K141, M145, H146, I147 and R148) were set as flexible in only one monomer of the dimer and docking was performed. Second, the same group of residues was set as flexible in both chains of the dimer. This setup was termed “full interface”. In both scenarios, two molecules of VD3 per dimer fit at the interface without steric overlap. Both molecules showed similar affinity despite the asymmetry induced by the “half interface” approach. The results also show a slight but clear affinity improvement as the total number of flexible residues increased (Table 2). That is, VD3 binds better when using the “full interface” setup. However, the difference between rigid vs. “full” flexible docking was just -0.8 kcal mol^-1^ (Table 2). This is a small improvement in binding affinity compared to the one attained by adding flexibility at the calyx, where the binding energy delta was greater than -3.0 kcal mol^-1^ for VD3, favoring the flexible setup over the rigid. This suggests that Site B is mostly formed by BLG dimerization with relatively minor contributions from side chain flexibility. It is noteworthy that our theoretical binding energy for VD3 favors Site A over Site B, even when using flexible residues (-9.1 vs -8.8 kcal mol^-1^, respectively Table 1 and 2), i.e. the calyx is energetically favored just as experimentally determined. Taken together, these data validate our setup for blind docking into the BLG dimer.

**Table 2:** Theoretical (docking) binding energies for vitamin D3 at the interfacial-binding site (site B) of dimeric 2BLG.

**Receptor**	Binding energy for Vitamin D3 (kcal mol^-1^)
Rigid Dimer	-8.0
Flexible Dimer (half interface)	-8.1
Flexible Dimer (full interface)	-8.8

### Lactose binding to bovine β-lactoglobulin in monomeric and dimeric form

While two binding sites for hydrophobic ligands have been clearly determined by biochemistry, crystallography and the theoretical results presented here (Figures 2 to 4), BLG's binding of small polar molecules such as lactose has been described as nonspecific or targeted to all exposed lysine residues [22]. When we performed lactose blind docking on the rigid 2BLG monomer, the three best results located to the same place: the previously mentioned Site C, with binding energies ranging between -6.0 and -6.4 kcal mol^-1^ (Table 3). Interestingly, lactose docking to a monomeric receptor with five or seven flexible calyx residues, favored the mouth of the calyx but didn't find Site A or Site C (Table 3). This probably arises from an overall enthalpy increase in the flexible setup that makes Site C less favorable. Since lactose did not locate the calyx and docking to the monomer resulted in low binding energies ranging from -5.4 to -6.4 kcal mol^-1^, we next explored the existence of lactose-binding sites in the BLG dimer. 

**Table 3:** Theoretical (docking) binding energies for lactose in monomeric and dimeric 2BLG.

**Receptor**	**Binding Site located**	**Binding energy (kcal mol^-1^)**
Rigid Monomer	Site C	-6.4
Monomer with flexible calyx ^a^	Calyx mouth	-5.4
Rigid Dimer	Sites B and C	-6.4
Dimer with flexible interface ^b^	Site B	-6.7

**Site C**. Non-interfacial site, located between sheet A and the last helix before the C-terminus.

**Site** B. Interfacial site.

^a^shows results with 7 flexible calyx residues. Equivalent results were obtained with 5 flexible residues.

^b^shows results for "full interface" docking, i.e. 7 flexible interfacial residues per subunit.

**Figure 5:**
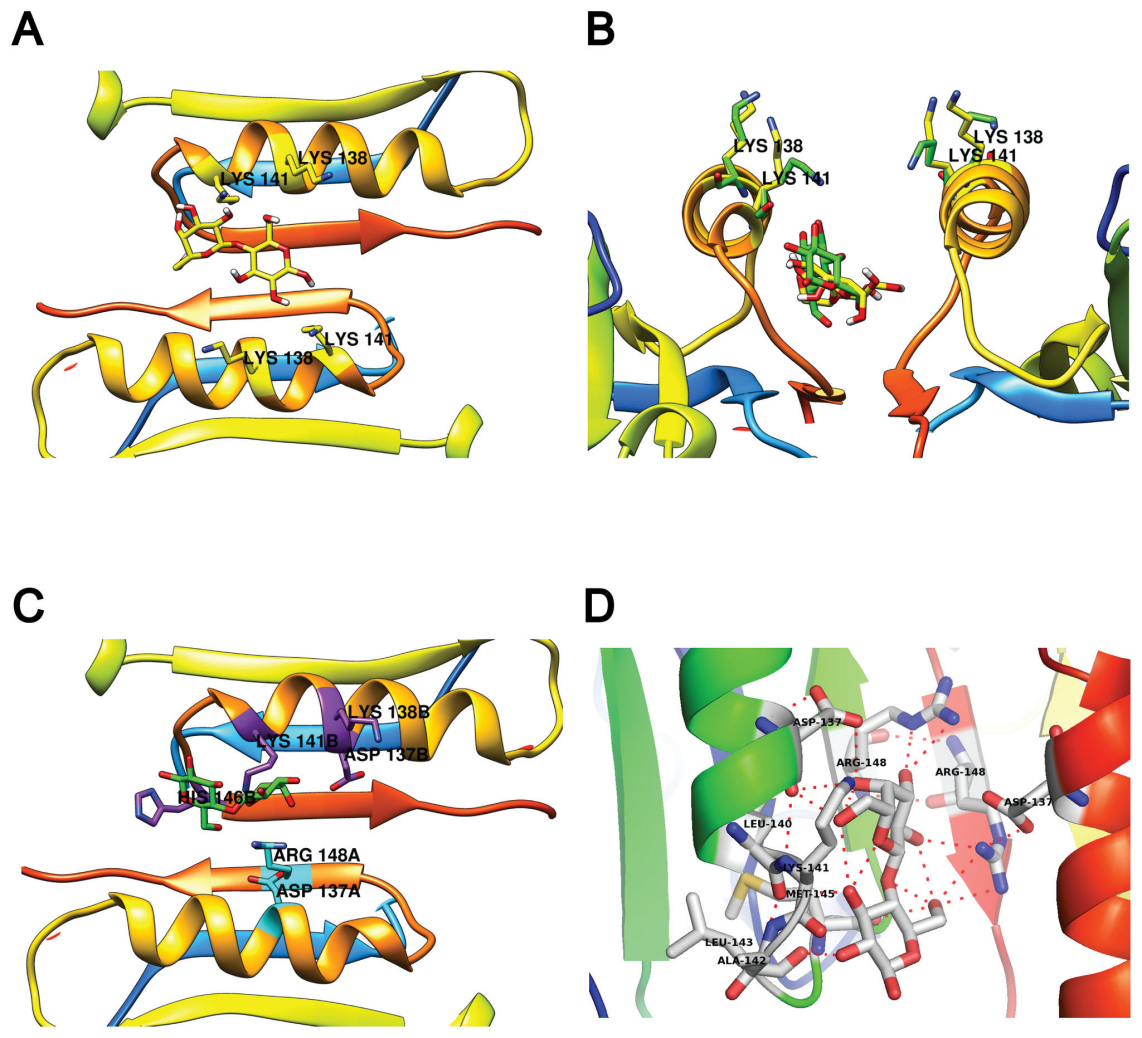
Lactose docking to the 2BLG dimer. (**A**) Lactose docking to a rigid BLG dimer. Notice both K138 and K141 pointing away from lactose. In (**B**), a side view of the rigid interface docking (lactose, K138 and K141 in yellow) is compared to the “fully flexible” results (green) where K141 shifts towards lactose. In (**C**) a top view of the best “fully flexible” result. Residues involved in lactose binding are highlighted by chain: chain B in purple and chain A in cyan. (**D**) close-up of the interfacial binding site showing chains from both BLG monomers and their respective hydrogen bonds to lactose.

Blind docking to the rigid 2BLG dimer detected Site B (the interface, Figure 5A) and Site C (Figure S3), but not Site A. Notably, the three best results were isoenergetic (Table 3) with two results favoring Site B and one Site C. Lactose docked in the rigid BLG dimer interface, was located 9.1 Å away from K47 in Site A (NZ to nearest sugar oxygen, Figure 5A) and 11.8 or 8.4 Å from K138 and K141 respectively in Site B, with side chains pointing away from the sugar. 

To test whether side chain flexibility affects lactose binding and its proximity to lysines, we followed the same strategy as for binding VD3 to the dimer. Results with the “half" and "full" interface were closely similar. Binding to a "full" flexible interface was favored over the rigid interface, by -0.3 kcal mol^-1^ (Table 3, Figure 5B). Close inspection showed that the residues binding lactose were K141 and H146 from one monomer and D137 and R148 from the other (Figure 5C). That is, the best binding results involve residues from both monomers. To delve further in this result, we ran docking tests using subsets of the “full interface” residues, that is, using less than the seven residues that bind VD3. This approach marginally increased the binding energy for lactose. The best binding energy with a set of 5 flexible interfacial residues per monomer was -6.8 kcal mol^-1^, with flexible D137, K141, H146, R148 and K138. K138 was added to test the possibility that repulsion between K138 and K141 influences their proximity to lactose. Notably, K141 flexibility allows its ε-amino to approach within 3.2 Å and 5.2 Å of the nearest sugar oxygen making it a good candidate for lactolation. As expected, when K141 is pointing towards lactose, K138 is always pointing away from the interface (Figure 5A and 5B). 

### Lactose binding to a flexible Site C

Since the binding energy differences between Site B and Site C are small, we tested whether a flexible Site C would bind lactose better than a flexible interface. Site C is composed of nine residues: Y20, Y42, E44, W59, L156, E157, E158, Q159 and H161. Binding energies for docking at Site C on a 2BLG monomer with these nine flexible residues yielded -6.6 kcal mol^-1^ (Figure S3), marginally better than to a rigid Site C. To avoid a large entropy increase due to the many degrees of freedom from the nine residues, we tested subsets of five flexible residues. Our best result was with E44, Q59, E157, E158 and Q159 and only improved binding energy to -6.7 kcal mol^-1^. The nearest lysine, K41, was located at about 11 Å. All approaches tested yielded results where lactose binds with similar energies to Site C and the interfacial Site B, although Site B was more populated and slightly more favorable. 

## Discussion

Although the biological function of BLG has not been fully established, it likely performs a role as a transporter for several ligands through the digestive tract. The interest and knowledge on this protein is well exemplified by the amount of structural work aimed to understand its binding mechanism. However, most of the techniques employed for determining its binding sites, such as mass spectrometry, require protein digestion or the loss of the dimeric form due to dilution. We chose blind and flexible docking as a novel approach to analyze, dissect and contrast the probable binding sites of the BLG monomer and dimer. We employed mainly 2BLG, an empty BLG structure with an open calyx lid (open EF loop), due to its model quality and absence of ligand bias. We validated this approach by comparing our docking results to XRD structures. Remarkably, only five flexible side chains in the calyx, out of the 23 that interact with ligands, are required to reproduce the calyx's experimental binding of diverse ligands (Table 1 and Figure 1C and 2D). Only VD3 needed two more flexible residues to bind the calyx. Yet, none of the ligands required a BLG dimer and Site B seems to be formed only in the dimeric BLG since VD3 docking to the monomer does not locate it. Moreover, the affinities found for 2BLG with flexible calyx residues are close to those determined experimentally and to those obtained from docking into 1B0O, a structure with a preformed binding site from co-crystallization with palmitic acid, where binding affinities do not further improve with side chain flexibility. Thus, we conclude that our system with side chain flexibility of selected residues allows the formation of the calyx-binding site in 2BLG and is useful not only to dissect known ligands but also to test for new ones. However, we are aware of the inherent limitations of our approach, namely, we cannot sample whole protein dynamics at side or main chain levels, or allow backbone flexibility. Probing of these movements during ligand binding will require a more complex approach, such as molecular dynamics, which has been successful to evaluate the opening/closing EF loop motion [38]. 

Overall, our analyses suggest that once the EF loop is open, BLG does not require extensive changes in the calyx's backbone to allow ligand binding, despite the various degrees of motion across its structure shown for example by NMR [44]. Nevertheless, our results are not at odds with data that relates the dynamics of BLG to ligand binding. In fact, they support a model for BLG calyx binding that can be summarized in three steps: 1. The EF loop opens in response to pH, with the consequential changes in the GH loop (as described by 12). This step is essential for binding. The ligand enters the calyx and successfully binds once a few side chains (L45, L54, I56, I71 and I84) undergo small rearrangements. This step gives rise to most of the binding energy (Figure 2D). 3. The ligand induces an overall change in protein dynamics [44], that likely contributes also to the binding energy.

In cow’s milk both lactose and BLG are very abundant, yet BLG's binding sites for lactose remain unknown. Our docking results with lactose show that it doesn't bind the calyx in our validated set up. However, lactose binds the dimer interface, at Site B, both when presented as rigid or as flexible. Adding flexibility to five or seven residues at the interface slightly increased lactose's affinity for Site B and brought NZ in K141 within 3.2 and 5.2 Å from the sugar. Furthermore, the best binding results show that residues from both subunits participate in lactose binding to the interface. Thus, lactose binding at the interface likely stabilizes the dimer and immobilizes the sugar within a claw-like structure so that a nucleophilic attack can occur leading to lactolation. K47 and K138 have been proposed in the past as the most likely residues to bind lactose due to their solvent exposure [22]. We recently reported evidence that pointed to lysines 138 or 141 as candidates for lactolation [20]. Our current study strongly points again to K141 as the first lysine to be lactolated. At high lactose concentration, other residues may follow. 

It is worth noting that, under most docking conditions, the interfacial Site B was preferred by lactose, although just slightly over Site C. Based on our results, we cannot discard Site C as a potential lactose-binding site. However, Site C cannot contribute to lactolation since no lysine is close enough to bound lactose at that site. To date there is no XRD or calorimetric evidence to support that Site C actually binds ligands, so the relevance and specificity of this site remains to be determined. Additionally, there is no proof that lactolation is necessary for lactose binding and our results could help design experiments to test this. 

It is important to emphasize the hydrophilic nature of Site B: molecular dynamics of dimeric BLG as well as isothermal titration calorimetry both have shown that dimer formation is accompanied by water sequestration to the interface [16]. That study also showed that dimer formation is a rigid body-like association [16]. Our results support this conclusion as they suggest that interfacial binding energies for both VD3 and lactose depend more on backbone than on side chain conformation. Taken together, these data point to the interface as a strong candidate for binding hydrophilic molecules such as lactose. Moreover, our data indicates that since lactose does not bind the calyx, both the calyx and the interface could be occupied at the same time by different ligands. To further validate our computational setup it will be important to confirm experimentally our predictions regarding lactose binding. 

Our results highlight the relevance of the BLG dimer as a robust template for protein redesign. Defining the minimal number of residues involved in binding to BLG is a major step forward to understand its mechanism for these and other ligands and move towards protein redesign. 

## Supporting Information

Figure S1 **Effect of loop conformation on calyx accessibility for BLG.** (**A**) And (**B**) show the 2BLG and 1BEB structures, respectively. When the EF loop is open (**A**) the calyx is accessible, but at low pH (< 6.0) (**B**) the EF loop closes over its entrance. (**C**) Shows the alignment of 14 structures used in this work with open EF loops, (empty/open 2BLG and the 13 ligand bound structures) showing the differences at loop I84-N90 (loop EF) and N109-S116 (loop GH).
(TIF)

Figure S2 **Pairwise RMSD by residue between structures 2BLG (open EF loop/empty) and (A) 1BSY (open EF loop/empty), (B) 3BLG (closed EF loop/empty) and (C) 1B0O, (open EF loop/palmitic acid-bound).**
(TIF)

Figure S3 **2BLG with lactose bound into Site C.** The best docking result obtained with nine flexible residues is shown. Similar results were found when docking was preformed against monomers or dimers. For clarity, only one BLG monomer is shown.
(TIF)

Figure S4 **Plot of binding energy from docking vs ligand** , **using the monomeric form of BLG, 1B0O, either rigid (black circles), or with 5 (black squares) or 7 (black triangles) flexible residues**.
Experimentally determined energies are shown for comparison (open circles). Fatty acids are sorted as in Figure 1C. PDB 1B0O contains palmitic acid, but the ligand was removed *in* *silico* before blind docking.
(TIF)

Table S1 **This table collects information about the number of contacts that each residue (leftmost column) establishes with the ligand (topmost row), in each XRD structure analyzed (PDB ID in bottom row).** Each colored square in the grid represents a contact of a residue with a ligand. Red squares were used for residues with at least 7 contacts with the ligand, blue for residues with 3 to 6 contacts and green for residues with 2 or less contacts. This grid illustrates the contact quantification used to selected residues for flexibility in the indicated dockings. The residues selected to set as flexible are depicted in white over a black square in the leftmost column.
(PDF)
