# Supplementary figures and images for: β-Lactoglobulin's Conformational Requirements for Ligand Binding at the Calyx and the Dimer Interphase: a Flexible Docking Study
Source: PLoS One. 2013 Nov 8;8(11):e79530. doi: 10.1371/journal.pone.0079530 (PMC3821863; doi:10.1371/journal.pone.0079530)

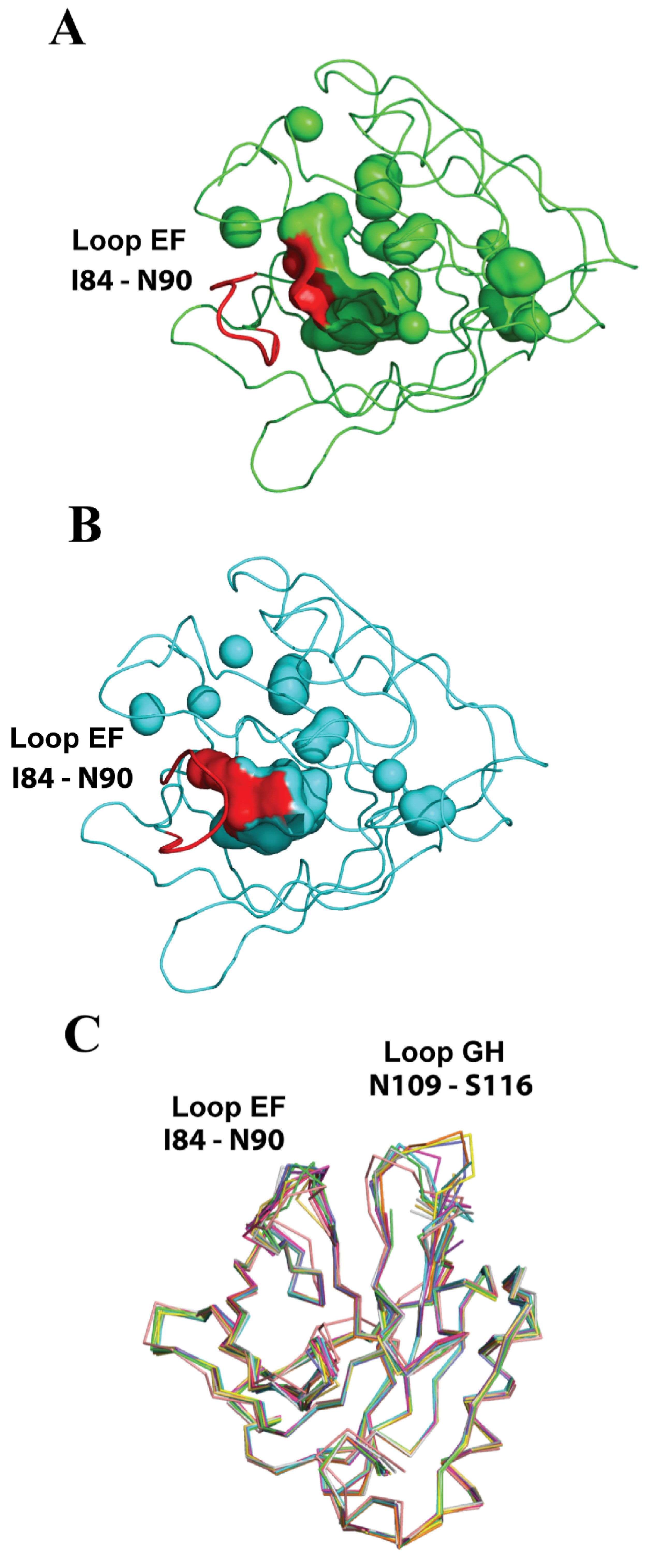

Supplement: Figure S1 — Effect of loop conformation on calyx accessibility for BLG. (A) And (B) show the 2BLG and 1BEB structures, respectively. When the EF loop is open (A) the calyx is accessible, but at low pH (< 6.0) (B) the EF loop closes over its entrance. (C) Shows the alignment of 14 structures used in this work with open EF loops, (empty/open 2BLG and the 13 ligand bound structures) showing the differences at loop I84-N90 (loop EF) and N109-S116 (loop GH). (TIF) [file pone.0079530.s001.tif]

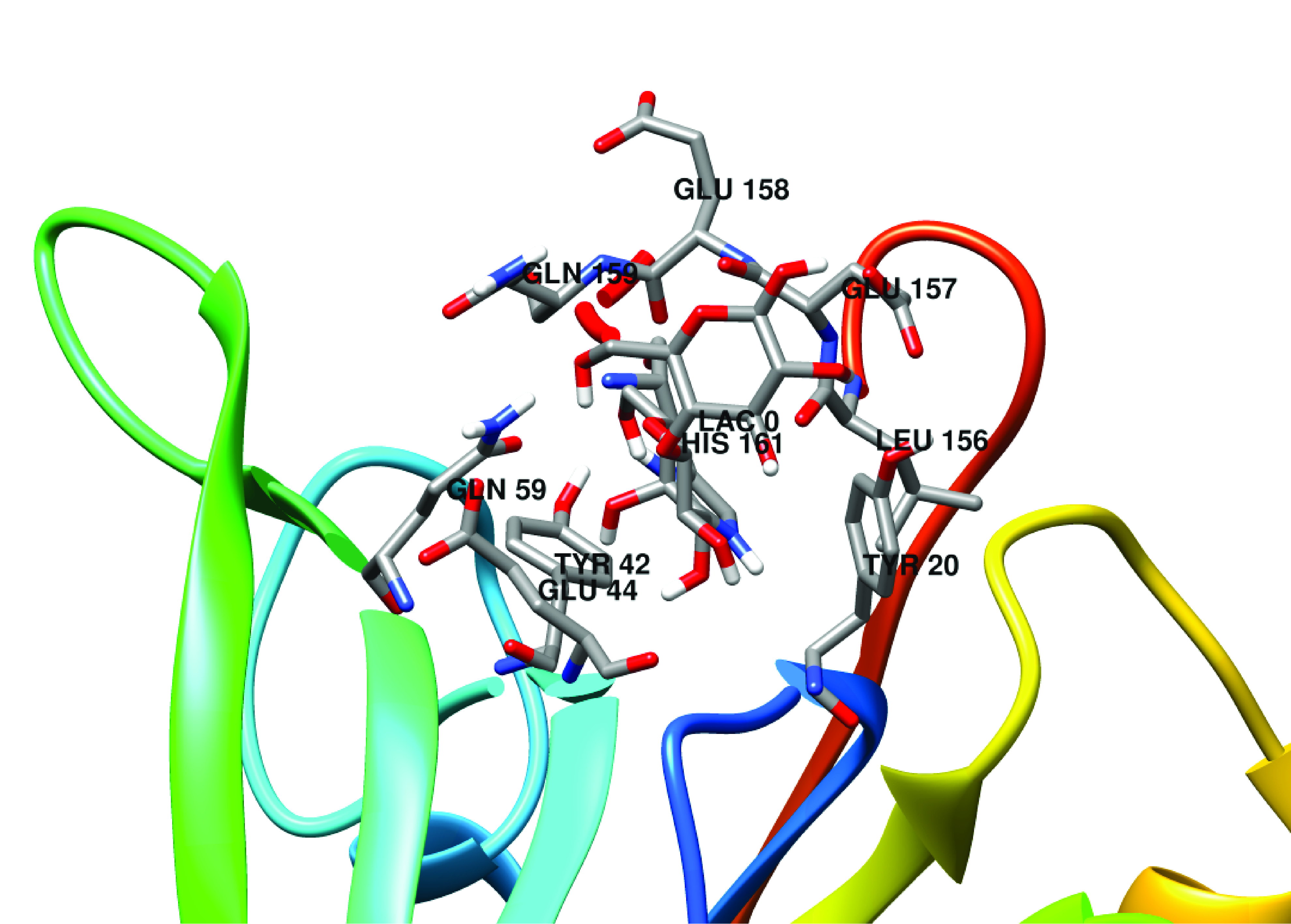

Supplement: Figure S3 — 2BLG with lactose bound into Site C. The best docking result obtained with nine flexible residues is shown. Similar results were found when docking was preformed against monomers or dimers. For clarity, only one BLG monomer is shown. (TIF) [file pone.0079530.s003.tif]
