# Supplementary material for: β-Lactoglobulin's Conformational Requirements for Ligand Binding at the Calyx and the Dimer Interphase: a Flexible Docking Study
Source: PLoS One. 2013 Nov 8;8(11):e79530. doi: 10.1371/journal.pone.0079530 (PMC3821863; doi:10.1371/journal.pone.0079530)
Supplement: Table S1 — This table collects information about the number of contacts that each residue (leftmost column) establishes with the ligand (topmost row), in each XRD structure analyzed (PDB ID in bottom row). Each colored square in the grid represents a contact of a residue with a ligand. Red squares were used for residues with at least 7 contacts with the ligand, blue for residues with 3 to 6 contacts and green for residues with 2 or less contacts. This grid illustrates the contact quantification used to selected residues for flexibility in the indicated dockings. The residues selected to set as flexible are depicted in white over a black square in the leftmost column. (PDF) [file pone.0079530.s005.pdf]

Supplemental Table 1. β-lg calyx-binding residues from the XRD structures.

	octanoic acid	decanoic acid	lauric acid	myristic acid	palmitic acid	Bromo-decanoic	linoleic acid	oleic acid	stearic acid	retinol	retinoic acid	Vitamin D3	Decyl Maltoside
I12									green				
P38				red	red		red	red	red			red	red
L39		blue								blue	blue	blue	
V41			blue	blue			blue	blue			blue	blue	
V43												green	
**L46**		red	red		red	red	red	red	red				
**L54**		red	red	red	red		red	red	red				
**I56**	red	red	red			red	red	red			red	red	red
L58				green									
K60					red	red	red	red	red			red	red
E62					blue		blue	blue		blue		blue	
K69	red			red	red	red	red			red	red		
**I71**	red	red		red	red	red	red	red	red	red	red	red	
**I84**		red	red	red	red	red		red	red			red	red
A86												green	green
L87													green
N88													green
V92	red		red			red	red	red	red	red	red		
V94		blue		blue	blue		blue	blue	blue				
L103			blue	blue				blue					blue
**F105**		red	red	red	red	red	red	red	red	red	red	red	red
**M107**	red	red	red	red	red	red	red	red	red	red	red	red	red
Q120										green	green		
PDB	***3NQ9***	***3NQ3***	***3UEU***	***3UEV***	***3UEW***	***1BSO***	***4DQ4***	***4DQ3***	***3UEX***	***1GX8***	***1GX9***	***2GJ5***	***2R56***

Color code is red for residues with at least 7 contacts with the ligand, blue for residues with 3 to 6 contacts and green for residues with 2 or less.
